# Retraction Note: Ultrasound biomicroscopy qualitative and quantitative signs of phacoantigenic uveitis

**DOI:** 10.1038/s41433-026-04458-5

**Published:** 2026-04-16

**Authors:** Francesco Pichi, Khalid Qadha, Khaled Almasri, Sahar AlAli, Piergiorgio Neri

**Affiliations:** 1 Eye Institute, Cleveland Clinic Abu Dhabi, Abu Dhabi, United Arab Emirates; 2 Cleveland Clinic Lerner College of Medicine, Case Western Reserve University, Cleveland, OH USA

**Keywords:** Biomarkers, Eye manifestations

Retraction Note to: *Eye* 10.1038/s41433-024-03317-5, published online13 September 2024

The Editor-in-Chief has retracted this article. Cleveland Clinic Abu Dhabi states that the ethics approval and consent forms from patients enrolled at Cleveland Clinic Abu Dhabi, as stated in this published article, were not obtained by the authors. The authors stated that they have enrolled patients from the Cleveland Clinic Abu Dhabi and San Giuseppe Clinic at the University of Milan. The authors provided an ethics approval document and consent forms for the study at the University of Milan; however, it shows that some patient samples were collected before ethics approval. Therefore, some of the patient samples in this study were collected unethically. The authors were unable to provide raw data and images from the patients enrolled San Giuseppe Clinic at the University of Milan.

The Editor-in-Chief, therefore, has lost confidence in the data provided in this article.

Francesco Pichi, Khaled Almasri, Piergiorgio Neri and Sahar AlAli disagree with this retraction. Khalid Qadha has not responded to any correspondence to the Publisher regarding this retraction.

